# Recursive Engine In-Cylinder Pressure Reconstruction Using Sensor-Fused Engine Speed

**DOI:** 10.3390/s24165237

**Published:** 2024-08-13

**Authors:** Runzhe Han, Christian Bohn, Georg Bauer

**Affiliations:** 1 College of Optical, Mechanical and Electrical Engineering, Zhejiang A&F University, Hangzhou 311300, China; 2 Institut für Elektrische Informationstechnik, Technische Universität Clausthal, 38678 Clausthal-Zellerfeld, Germany; bohn@iei.tu-clausthal.de (C.B.); bauer@iei.tu-clausthal.de (G.B.)

**Keywords:** sensor fusion, Kalman filters, engine in-cylinder pressure reconstruction, virtual sensing

## Abstract

The engine in-cylinder pressure is a very important parameter for the optimization of internal combustion engines. This paper proposes an alternative recursive Kalman filter-based engine cylinder pressure reconstruction approach using sensor-fused engine speed. In the proposed approach, the fused engine speed is first obtained using the centralized sensor fusion technique, which synthesizes the information from the engine vibration sensor and engine flywheel angular speed sensor. Afterwards, with the fused speed, the engine cylinder pressure signal can be reconstructed by inverse filtering of the engine structural vibration signal. The cylinder pressure reconstruction results of the proposed approach are validated by two combustion indicators, which are pressure peak $P_{\max}$ and peak location $P_{\mathrm{loc}}$. Meanwhile, the reconstruction results are compared with the results obtained by the cylinder pressure reconstruction approach using the calculated engine speed. The results of sensor fusion can indicate that the fused speed is smoother when the vibration signal is trusted more. Furthermore, the cylinder pressure reconstruction results can display the relationship between the sensor-fused speed and the cylinder pressure reconstruction accuracy, and with more belief in the vibration signal, the reconstructed results will become better.

## 1. Introduction

The reconstruction of the in-cylinder pressure of internal combustion engines plays an important role in optimizing monitoring and control systems in order to ensure combustion efficiency, decrease harmful emissions, and detect possible engine faults [1,2,3,4,5]. Direct measurements provided by engine in-cylinder pressure transducers are obviously a feasible solution; see for instance [6]. However, in addition to experimental studies, this is unusual in production engines due to the following factors: difficult installation, long-term reliability issues, and cost limitations.

Considering the disadvantages of the direct use of cylinder pressure transducers, numerous cylinder pressure reconstruction approaches without cylinder pressure transducers or with only one cylinder pressure transducer have been proposed, and in general, there are three types of cylinder pressure reconstruction approaches:

(1) Solely vibration-based reconstruction
  A fast pressure change in a cylinder during combustion leads to engine structural vibrations, which indicate that the engine structural vibration signal contains information related to the combustion process such that it has the potential to recover the cylinder pressure signal [7,8,9]. With the relationship between cylinder pressure and vibration, various cylinder pressure reconstruction approaches have been proposed, such as frequency response function-based approaches [10,11,12,13] and artificial neural network-based approaches [14,15].
(2) Solely crank speed-based reconstruction
  The engine crank speed fluctuation versus crank angle contains information about the cylinder-by-cylinder combustion pressure [16]. Many researchers have explored the relationship between speed fluctuation and cylinder pressure. In [17], the researchers made a model of the cylinder pressure signal using the crank angular speed from a statistical point of view. In [18], the researchers used the frequency response function between the cylinder pressure and the crank angular speed converted by a time-domain model and applied frequency response function mapping to improve the accuracy of cylinder pressure reconstruction under time-variant operating points. Based on the engine energy model, in [19,20], the authors used the extended sliding observer and the Kalman filter, respectively, to reconstruct the cylinder pressure. In [21], a single-cylinder pressure sensor and a crank angle sensor were used to reconstruct the cylinder pressure for a six-cylinder heavy-duty diesel engine. In addition, approaches like artificial neural networks have also been investigated [15,22,23,24,25].
(3) Combination of vibration and crank speed-based reconstruction
  It has been shown that both engine structural vibration and crank angular speed contain information about the cylinder pressure but mainly in different frequency regions [16]. In [16], the cylinder pressure was reconstructed based on complex-valued radial basis function network using both vibration and speed signal. In [26], a recursive engine in-cylinder pressure reconstruction approach by the use of both engine vibration and engine speed was proposed.

Among the above cylinder pressure reconstruction approaches, the cylinder pressure reconstruction approach proposed in [26] has the following advantages:

(1) Problems regarding spectrum leakage, ill-conditioned inversion, and frequency response function variations do not exist.
(2) It avoids the training of networks such that large amounts of data are not necessary.
(3) It does not depend on the engine energy model. Building the engine energy model can be expensive and time-consuming in practice, especially when the model is used for different types of engines.
(4) It completely eliminates the need for a physical cylinder pressure transducer.

In [26,27], excluding the engine structural vibration signal, the engine speed is required. However, merely using the conditioned pulse signal from the angular position sensor, the engine speed calculation may involve two types of errors: spectral aliasing and quantization error [28], so the accuracy of the cylinder pressure reconstruction could be affected. As the vibration signal contains information related to the engine speed [16], sensor fusion approaches [29] merging the information from both vibration sensor and engine speed sensor could be implemented to obtain a fused speed [30]. Afterwards, if the fused speed takes the place of the calculated engine speed in [26] to reconstruct the cylinder pressure signal, the reconstruction accuracy may be enhanced.

With the above idea, this paper proposes a sensor-fused engine speed-based cylinder pressure reconstruction approach, and the main contributions of this paper are as follows:

(1) A sensor fusion-based engine speed estimation approach is proposed.
(2) The accuracy of the cylinder pressure reconstruction can be enhanced by tuning the weightings in the sensor fusion of the engine speed.

The remaining parts of the paper are organized as follows. In Section 2, the engine test bench used for validating cylinder pressure reconstruction results is demonstrated, and in Section 3, the approach of calculating the engine speed based on using an engine flywheel angular position sensor is introduced, while Section 4 illustrates how the engine speed can be derived using an accelerometer. In Section 5, the engine speed is obtained by fusing the data from the engine flywheel angular position sensor and the vibration sensor, based on which Section 6 proposes the cylinder pressure reconstruction approach by using the sensor fusion technique. In Section 7, experimental studies are conducted; the results show the relationship between sensor-fused speed and cylinder pressure reconstruction accuracy and show the comparison results between the proposed approach in this paper and the approach proposed in [26]. Finally, conclusions and perspectives are provided in Section 8.

## 2. Test Bench

**Figure 1:**
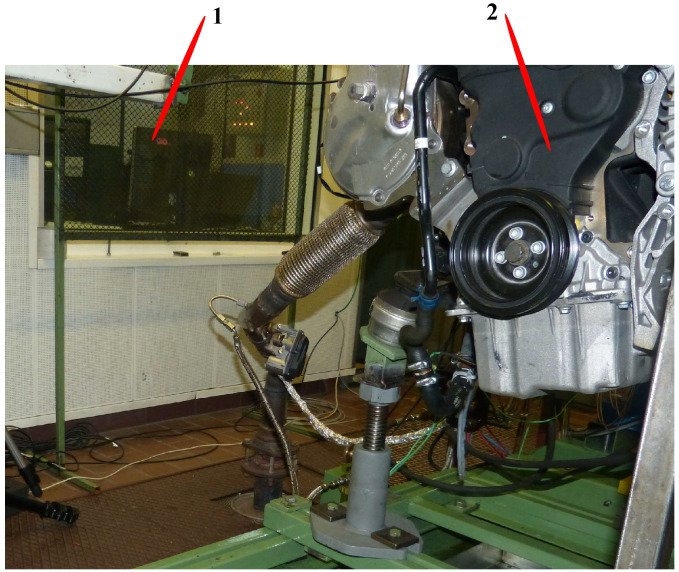
Engine test bench (1: control and measuring system; 2: Volkswagen 2.0 TDI engine with four-stroke cycle and four cylinders).

Figure 1 displays the engine test bench used for the validation of the proposed engine cylinder pressure reconstruction approach. The engine is the Volkswagen 2.0 TDI engine, which has four strokes and four cylinders. In the engine, the flywheel angular position sensor, the vibration sensor, and four cylinder pressure sensors are installed to measure the crank angular speed, the vibration, and the cylinder pressure, respectively. The flywheel angular position sensor (a Hall sensor) is installed close to the flywheel, the vibration sensor is mounted on the outer wall of the internal combustion engine, and the cylinder pressure sensor is installed in the cylinders. In the test bench, the cylinder pressure, the crank angular speed, and the vibration signal (acceleration) can be collected synchronously. The cutoff frequency and the sampling frequency were chosen as 1 kHz and 20 kHz, respectively.

In the paper, dataset Z collected from the test bench represents the process from the engine operating condition 2100 rpm and 180 Nm to the engine operating condition 3000 rpm and 60 Nm. Dataset Z is used to validate the proposed approach. The operating conditions in the dataset Z are consecutive.

## 3. Engine Speed Sensing by a Flywheel Angular Position Sensor

The signal collected from the flywheel angular position sensor installed in the test bench cannot be directly implemented in the proposed cylinder pressure reconstruction approach because the pulse signal should be changed to the speed in rad/s or in Hz.

**Figure 2:**
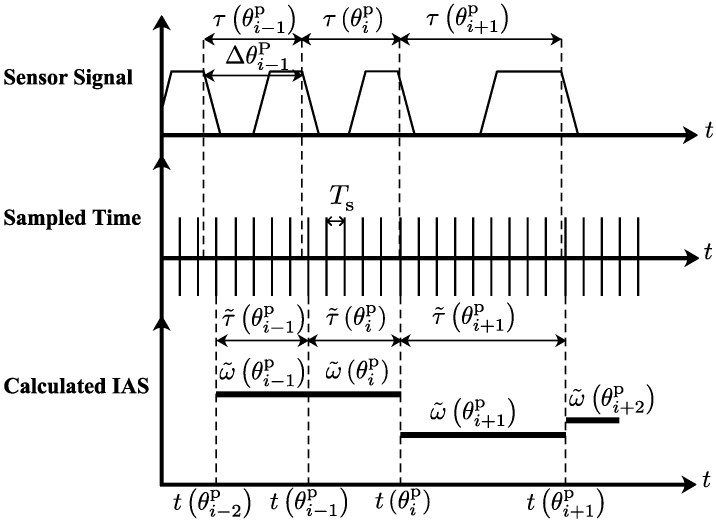
Instantaneous angular speed calculation (*t*: time; θ: crank angle; *i*: integral number; wider pulse: corresponds to the reference marker of the engine flywheel; IAS: instantaneous angular speed).

The principle behind the calculation of the instantaneous angular speed $\tilde{\omega }$ in rad/s is simply illustrated in Figure 2. Below, take *i*-th $\Delta \theta^{p}$ (i.e., $\Delta \theta_{i}^{p}$) as an example. The Hall-effect sensor first collects the flywheel angular position signal, then the instantaneous angular speed signal $\tilde{\omega }\left(\theta_{i}^{p}\right)$ can be calculated as follows:

(1) $$\tilde{\omega }\left(\theta_{i}^{p}\right)\approx \frac{\Delta \theta_{i}^{p}}{\tilde{\tau }\left(\theta_{i}^{p}\right)},$$

where $\theta_{i}^{p}$ denotes the angle obtained by the flywheel angular position sensor, $\Delta \theta_{i}^{p}$ denotes the angle between two consecutive falling edges, $\Delta \theta_{i}^{p}$ is $6^{\circ }$ (and $12^{\circ }$ for the reference marker), and $\tilde{\tau }\left(\theta_{i}^{p}\right)$ denotes the value to approximate the elapsed time $\tau (\theta_{i}^{p})$ between two consecutive falling edges.

The relationship between $\tilde{\omega }\left(k\right)$ and $\tilde{\omega }\left(\theta_{i}^{p}\right)$ is as follows:

(2) $$\tilde{\omega }\left(k\right)=\tilde{\omega }\left(\theta_{i}^{p}\right)\;\mathrm{for}\;k=\frac{\theta_{i-1}^{p}}{T_{s}},\ldots ,\frac{\theta_{i}^{p}}{T_{s}},$$

where $T_{s}$ denotes the sampling period which is the inverse of the sampling frequency 20 kHz.

The cylinder pressure reconstruction algorithm needs the instantaneous engine cycle frequency f(k) in Hz (each engine cycle, $720^{\circ }$), and the value of instantaneous engine cycle frequency is half of the instantaneous engine angular speed in revolutions per second. The frequency f(k) can be calculated as follows:

(3) $$f\left(k\right)\approx \frac{{\tilde{\omega }}_{d}\left(k\right)}{4\pi },$$

where ${\tilde{\omega }}_{d}\left(k\right)$ represents the delayed version of the calculated instantaneous angular speed $\tilde{\omega }\left(k\right)$, and the speed ${\tilde{\omega }}_{d}\left(k\right)$ can be calculated as follows:

(4) $${\tilde{\omega }}_{d}\left(k\right)=\tilde{\omega }\left(\theta_{i}^{p}\right)\;\mathrm{for}\;k=t_{s}\left(k\right),\ldots ,t_{e}\left(k\right),$$

where $t_{s}\left(k\right)=\mathrm{find}\left(t\left(k\right)\geq t\left(\theta_{i-1}^{p}\right),1\right)+1$, and $t_{e}\left(k\right)=\mathrm{find}\left(t\left(k\right)\geq t\left(\theta_{i}^{p}\right),1\right)$. “find” denotes the MATLAB function.

The reader can be referred to [28] for more details about several factors affecting the precision of the calculated instantaneous angular speed.

## 4. Engine Speed Sensing by a Vibration Sensor

Under stationary operating conditions, the engine structural vibration signal y(k)∈R can be approximately considered as a summation of a certain number of spectral components [31], so the vibration signal y(k) can be represented as the output of the following state-space model [32]:

(5) $$\left\{\begin{array}{cc} x_{y}\left(k+1\right) & =A_{y}\left(f\left(k\right)\right)x_{y}\left(k\right),\\ y(k) & =C_{y}x_{y}\left(k\right)+v_{y}\left(k\right), \end{array}\right.$$

where the state vector $x_{y}\left(k\right)\in R^{2n_{y}+1}$ denotes the state vector, the term $v_{y}\left(k\right)$ denotes the output error, and the state matrix $A_{y}\left(k\right)$ and the output matrix $C_{y}$ are given as follows:

(6) $$A_{y}\left(f\left(k\right)\right)=\left(\begin{array}{cccc} 1 & 0 & \cdots & 0\\ 0 & A_{1}\left(f\left(k\right)\right) & \ddots & \vdots \\ \vdots & \ddots & \ddots & 0\\ 0 & \cdots & 0 & A_{n_{y}}\left(f\left(k\right)\right) \end{array}\right),$$

and

(7) $$C_{y}=\left(\begin{array}{cccc} 1 & C_{1} & \cdots & C_{n_{y}} \end{array}\right),$$

respectively.

In the matrices $A_{y}\left(f\left(k\right)\right)$ and $C_{y}$, the individual block entries are denoted as follows:

(8) $$A_{i}\left(f\left(k\right)\right)=\left(\begin{array}{cc} \cos(2\pi if\left(k\right)T_{s}) & \sin(2\pi if\left(k\right)T_{s})\\ -\sin(2\pi if\left(k\right)T_{s}) & \cos(2\pi if\left(k\right)T_{s}) \end{array}\right)$$

and

(9) $$C_{i}=\left(\begin{array}{cc} 1 & 0 \end{array}\right).$$

Because only the first order of the signal y(k) is of interest, a bandpass filter can be used to filter out the orders that are not of interest in the signal y(k), such that the dimension of the model (5) can be decreased. The filtered y(k) is denoted as $y_{b}\left(k\right)$. For the purpose of tracking the frequency f(k), it is enough for us to estimate the frequency f(k) by only knowing the first order of the signal y(k). For the implementation of the bandpass filter, it is not necessary to guarantee the linear phase because the objective is to merely estimate the frequency f(k). Thus, IIR filters can be used instead of FIR filters; in addition, big dimensional problems can be avoided if IIR filters are used [33]. A Butterworth bandpass filter can be seen as a choice. The filtered signal $y_{b}\left(k\right)$ can be represented as the output of the following state-space model with an output error:

(10) $$\left\{\begin{array}{cc} x_{b}\left(k+1\right) & =A_{b}\left(f\left(k\right)\right)x_{b}\left(k\right),\\ y_{b}\left(k\right) & =C_{b}x_{b}\left(k\right)+v_{b}\left(k\right), \end{array}\right.$$

where the matrices $A_{b}\left(f\left(k\right)\right)$ and $C_{b}$ are denoted as follows:

(11) $$A_{b}\left(f\left(k\right)\right)=\left(\begin{array}{cc} A_{1}\left(f\left(k\right)\right) & 0\\ 0 & A_{2}\left(f\left(k\right)\right) \end{array}\right)$$

and

(12) $$C_{b}=\left(\begin{array}{cc} C_{1} & C_{2} \end{array}\right),$$

respectively, and the term $v_{b}\left(k\right)$ denotes the output error after filtering.

We replace the variable f(k) in the model (10) with the state variable $x_{f}\left(k\right)$, and then augment $x_{f}\left(k\right)$ with $x_{b}\left(k\right)$ such that the following model can be obtained:

(13) $$\left\{\begin{array}{cc} \left(\begin{array}{c} x_{b}\left(k+1\right)\\ x_{f}\left(k+1\right) \end{array}\right) & =\left(\begin{array}{cc} A_{b}\left(x_{f}\left(k\right)\right) & 0\\ 0 & 1 \end{array}\right)\left(\begin{array}{c} x_{b}\left(k\right)\\ x_{f}\left(k\right) \end{array}\right),\\ y_{b}\left(k\right) & =\left(\begin{array}{cc} C_{b} & 0 \end{array}\right)\left(\begin{array}{c} x_{b}\left(k\right)\\ x_{f}\left(k\right) \end{array}\right)+v_{b}\left(k\right). \end{array}\right.$$

The state-space model (13) can be changed into the following form:

(14) $$\left\{\begin{array}{cc} x_{e}\left(k+1\right) & =f_{e}\left(x_{e}\left(k\right)\right),\\ y_{b}\left(k\right) & =C_{e}x_{e}\left(k\right)+v_{b}\left(k\right). \end{array}\right.$$

In the model (14), the expressions of $x_{e}\left(k\right)$, $f_{e}\left(x_{e}\left(k\right)\right)$, and $C_{e}$ are

(15) $$x_{e}\left(k\right)=\left(\begin{array}{c} x_{b}\left(k\right)\\ x_{f}\left(k\right) \end{array}\right),$$

(16) $$f_{e}\left(x_{e}\left(k\right)\right)=\left(\begin{array}{cc} A_{b}\left(x_{f}\left(k\right)\right) & 0\\ 0 & 1 \end{array}\right),$$

and

(17) $$C_{e}=\left(\begin{array}{cc} C_{b} & 0 \end{array}\right),$$

respectively.

With the nonlinear model (14) and the filtered vibration signal $y_{b}\left(k\right)$, the extended Kalman filter can be implemented to estimate the state variable $x_{f}\left(k\right)$ [34,35], and the estimate ${\hat{x}}_{f}\left(k\right)$ can be seen as the estimated value of the instantaneous engine cycle frequency f(k), i.e., ${\hat{x}}_{f}\left(k\right)=\hat{f}\left(k\right)$. The symbol “^” denotes the estimate or reconstructed value.

It should be noted that the formulation of the model (14) is based on the assumption that both the amplitude of the first order of the signal $y_{b}\left(k\right)$ and the frequency f(k) are time-invariant. Thus a forgetting factor should be involved in the extended Kalman filter for the model (14) to cope with time-variant issues [35]. Furthermore, in the model (14), $\left\{v_{b}\left(k\right)\right\}$ is assumed to be white noise process, of which the covariance function is $R_{b}\delta_{kj}$ with $\delta_{kj}$ the Kronecker Delta function, and the value of $R_{b}$ is tunable.

**Figure 3:**
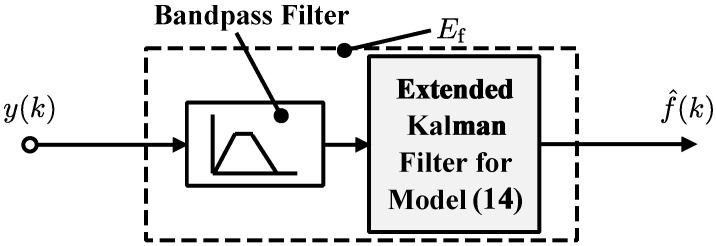
Proposed estimator $E_{f}$ for frequency tracking.

The above frequency estimation process can be realized by a frequency estimator $E_{f}$, which consists of a bandpass filter and the extended Kalman filter. The frequency estimator $E_{f}$ is depicted in Figure 3.

Because in the matrix $A_{b}\left(x_{f}\left(k\right)\right)$ in (14) there are two blocks, $A_{1}\left(x_{f}\left(k\right)\right)$ and $A_{2}\left(x_{f}\left(k\right)\right)$, which correspond to f(k) and 2f(k), respectively, in the extended Kalman filter, the initial value of the frequency f(k) should be chosen correctly.

Below is an example to explain the above initialization problem; if the real value of the instantaneous engine cycle frequency to be estimated is 25 Hz, the initial value of the frequency is chosen as 10 Hz in the extended Kalman filter, and the estimated instantaneous engine cycle frequency will be around 12.5 Hz which is not correct. Therefore, the algorithm for the engine cycle frequency estimation should involve a strategy to solve the above problem.

Above all, the vibration sensor-based engine speed estimation algorithm is summarized in Algorithm 1.

**Algorithm 1:** Vibration sensor-based engine cycle frequency estimation algorithm.

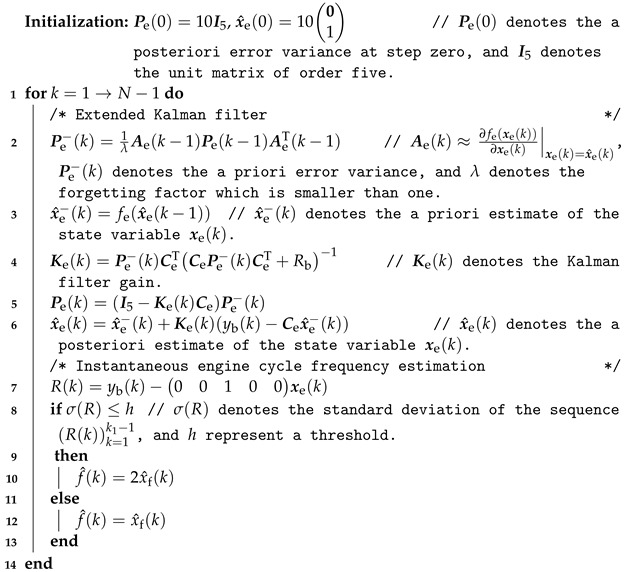



As seen at the end of Algorithm 1, a strategy to handle the initialization problem is implemented. Specifically, we first observe the FFT spectrums of the value of R(k) under different engine operating conditions, then set a value for the threshold *h*. Based on the threshold *h*, the instantaneous engine cycle frequency can be tracked correctly.

## 5. Sensor Fusion-Based Engine Speed Estimation

**Figure 4:**
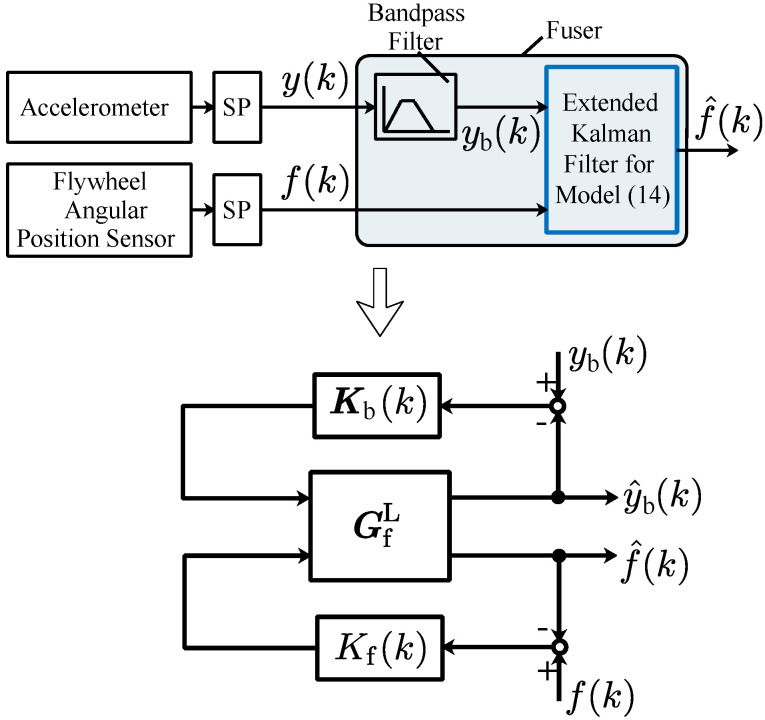
Centralized sensor fusion (SP: signal processing; $G_{f}^{L}$: the linearized model of the nonlinear model $G_{f}$; $K_{b}\left(k\right)$ and $K_{f}\left(k\right)$: the Kalman filter gains).

In Section 3 and Section 4, the instantaneous engine cycle frequency can be obtained using the flywheel angular position sensor and the vibration sensor, respectively. By merging the information from the vibration sensor and the flywheel angular position sensor, the fused instantaneous engine cycle frequency can be obtained. Generally, there are two kinds of sensor fusion approaches: one is the centralized sensor fusion approach, and the other one is the decentralized sensor fusion approach [29]. Compared with the decentralized sensor fusion approach, the structure of the algorithm of the centralized sensor fusion approach is more compact, so here the centralized sensor fusion approach is used to correlate and fuse the data from the vibration sensor and flywheel angular position sensor. The idea of how to implement the sensor fusion approach is shown in Figure 4.

Below, the specific steps for the realization of the centralized sensor fusion in Figure 4 are illustrated.

Step 1: Augment the output $y_{b}\left(k\right)$ in the model (14) with the instantaneous engine cycle frequency f(k), then the following nonlinear model $G_{f}$ can be obtained:
  (18) $$\left\{\begin{array}{cc} \left(\begin{array}{c} x_{b}\left(k+1\right)\\ x_{f}\left(k+1\right) \end{array}\right) & =\left(\begin{array}{cc} A_{b}\left(x_{f}\left(k\right)\right) & 0\\ 0 & 1 \end{array}\right)\left(\begin{array}{c} x_{b}\left(k\right)\\ x_{f}\left(k\right) \end{array}\right),\\ \left(\begin{array}{c} y_{b}\left(k\right)\\ f(k) \end{array}\right) & =\left(\begin{array}{cc} C_{b} & 0\\ 0 & 1 \end{array}\right)\left(\begin{array}{c} x_{b}\left(k\right)\\ x_{f}\left(k\right) \end{array}\right)+\left(\begin{array}{c} v_{b}\left(k\right)\\ v_{f}\left(k\right) \end{array}\right), \end{array}\right.$$
  where the term $v_{f}\left(k\right)$ represents the output error induced by the difference between f(k) and $x_{f}\left(k\right)$.
Step 2: Obtain the extended Kalman filter for the model (18), such that the instantaneous engine cycle frequency can be estimated. The extended Kalman filter can be seen as the fuser in the sensor fusion approach illustrated in Figure 4. The specific sensor fusion-based instantaneous engine cycle frequency estimation algorithm is summarized in Algorithm 2.
  In Algorithm 2,
  (19) $$x_{e}\left(k\right)=\left(\begin{array}{c} x_{b}\left(k\right)\\ x_{f}\left(k\right) \end{array}\right),$$
  and
  (20) $$y_{e}\left(k\right)=\left(\begin{array}{c} y_{b}\left(k\right)\\ f(k) \end{array}\right),$$
  and
  (21) $$C_{e}^{*}=\left(\begin{array}{cc} C_{b} & 0\\ 0 & 1 \end{array}\right),$$
  and
  (22) $$K_{e}^{*}\left(k\right)=\left(\begin{array}{c} K_{b}\left(k\right)\\ K_{f}\left(k\right) \end{array}\right),$$
  and $K_{b}\left(k\right)$ and $K_{f}\left(k\right)$ represent the Kalman filter gains corresponding to $y_{b}\left(k\right)$ and f(k), respectively, and
  (23) $$f_{e}\left(x_{e}\left(k\right)\right)=\left(\begin{array}{cc} A_{b}\left(x_{f}\left(k\right)\right) & 0\\ 0 & 1 \end{array}\right)\left(\begin{array}{c} x_{b}\left(k\right)\\ x_{f}\left(k\right) \end{array}\right).$$
  Furthermore, in Algorithm 2, the matrix Q and the matrix R denote the covariance matrix of the white noise $w_{e}\left(k\right)$ and the covariance matrix of the white noise $v_{e}\left(k\right)$, respectively. $w_{e}\left(k\right)$ and $v_{e}\left(k\right)$ represent the process noise and the measurement noise, respectively, of the following state-space model:
  (24) $$\left\{\begin{array}{cc} x_{e}^{*}\left(k+1\right) & =f_{e}\left(x_{e}^{*}\left(k\right)\right)+w_{e}\left(k\right),\\ \left(\begin{array}{c} v_{b}\left(k\right)\\ v_{f}\left(k\right) \end{array}\right) & =C_{e}^{*}x_{e}^{*}\left(k\right)+v_{e}\left(k\right), \end{array}\right.$$
  where the vector $x_{e}^{*}\left(k\right)$ denotes the state vector which has the same dimension as the state vector $x_{e}\left(k\right)$.
  The matrix Q and matrix R are set to be a matrix in a diagonal form, i.e.,
  (25) $$Q=QI_{5},$$
  and
  (26) $$R=\left(\begin{array}{cc} R_{1} & 0\\ 0 & R_{2} \end{array}\right),$$
  where *Q* denotes a real number, $I_{5}$ denotes the unit matrix of order five, and $R_{1}$ and $R_{2}$ correspond to $y_{f}\left(k\right)$ and f(k), respectively. By tuning the values of Q and R, we can indirectly tune the gains $K_{b}\left(k\right)$ and $K_{f}\left(k\right)$, which is equivalent to tuning the weightings on $x_{y}\left(k\right)$, $x_{f}\left(k\right)$, $y_{f}\left(k\right)$, and f(k) [36], e.g., we can tune R to make a decision on which sensor (flywheel angular position sensor or vibration sensor) should be more believed. So, we can finally obtain different estimates of the instantaneous engine cycle frequency, with which we can check the cylinder pressure reconstruction results variations.

**Algorithm 2:** Sensor fusion-based instantaneous engine cycle frequency estimation.

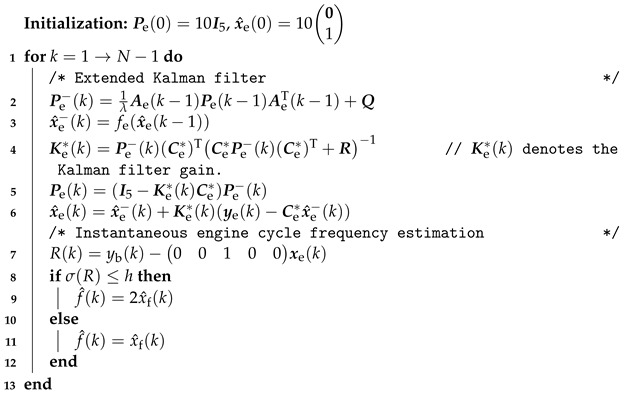



## 6. Sensor-Fused Engine Speed-Based Cylinder Pressure Reconstruction

In this section, the calculated speed-based cylinder pressure reconstruction approach derived in [26] is introduced first. Then, the sensor fusion-based cylinder pressure reconstruction approach is proposed.

### 6.1. Calculated Speed-Based Cylinder Pressure Reconstruction

**Figure 5:**
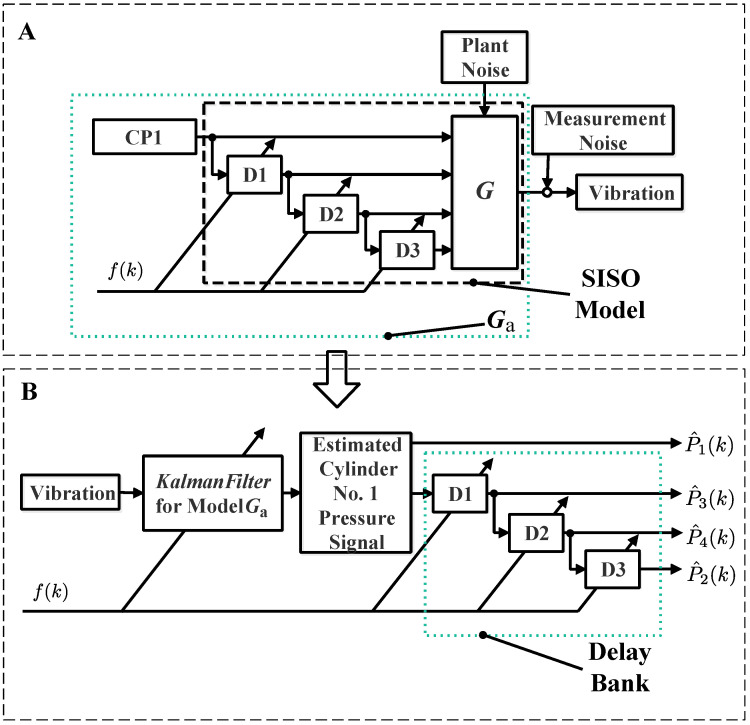
Calculated speed-based cylinder pressure reconstruction approach (CP1: cylinder No. 1 pressure signal model; D1, D2, D3: delay block).

As illustrated in Figure 5, the total framework in [26] contains two parts: part A and part B.

Part A can be seen as an offline design process which consists of three steps:

(i) Use system identification approaches to identify a model G between cylinder pressure and vibration. The model G is a discrete time, linear, time-invariant model, which has four inputs and one output.
(ii) Use a delay bank containing three delay blocks to make other three cylinder pressure curves be the delayed curves of the cylinder No. 1 pressure curve.
(iii) Obtain the augmented model $G_{a}$ by connecting three models, i.e., the cylinder pressure signal model, the model G, and three delay blocks.

While part B can be seen as an online implementation process. Specifically, a linear Kalman filter for the augmented model $G_{a}$ can be first derived, and then the vibration signal can be used to reconstruct the value of the cylinder No. 1 pressure signal recursively. Based on the delay block bank, the other three cylinder pressure signals can be reconstructed simultaneously.

Below, part A and part B are illustrated step by step with mathematical descriptions.

Step 1: Use system identification approaches to identify the model between cylinder pressure and vibration signal, and denote the identified model as $\hat{G}$. The model $\hat{G}$ has four inputs (i.e., four cylinder pressure signals $P_{m}\left(k\right)\in R$, m=1,2,3,4) and one output (i.e., the vibration signal y(k)). The state-space representation of the model $\hat{G}$ is as follows:
  (27) $$\left\{\begin{array}{cc} x(k+1) & =Ax(k)+Bu(k),\\ y(k) & =Cx\left(k\right)+Du\left(k\right)+v_{g}\left(k\right), \end{array}\right.$$
  where $x\left(k\right)\in R^{n}$ denotes the state vector, the matrices A, B, C, and D denote the state matrix, the input matrix, the output matrix, and the feedthrough matrix, respectively, the term $v_{g}\left(k\right)$ denotes the output error, and the input $u\left(k\right)\in R^{4}$ is represented as follows:
  (28) $$u\left(k\right)=\left(\begin{array}{c} P_{1}\left(k\right)\\ P_{2}\left(k\right)\\ P_{3}\left(k\right)\\ P_{4}\left(k\right) \end{array}\right).$$
Step 2: Under stationary engine operating conditions, similar to the modeling of the vibration signal in Equation (5), the cylinder pressure signal $P_{1}\left(k\right)$ can be expressed as the output of the following state-space model:
  (29) $$\left\{\begin{array}{cc} x_{p}\left(k+1\right) & =A_{p}\left(f\left(k\right)\right)x_{p}\left(k\right),\\ P_{1}\left(k\right) & =C_{p}x_{p}\left(k\right)+v_{p}\left(k\right), \end{array}\right.$$
  where the vector $x_{p}\left(k\right)\in R^{n_{p}}$ denotes the state vector, $v_{p}\left(k\right)$ represents the output error, the value of $v_{p}\left(k\right)$ should be guaranteed to be a small value, and the matrix $A_{p}\left(f\left(k\right)\right)$ and the matrix $C_{p}$ denote the state matrix and the output matrix, respectively.
Step 3: As displayed in Figure 5, D1, D2, and D3 denote the symbols of three single-input, single-output delay blocks. The model of the each delay block can be expressed as follows:
  (30) $$\left\{\begin{array}{cc} x_{d}\left(k+1\right) & =G_{d}\left(f\left(k\right)\right)x_{d}\left(k\right)+H_{d}\left(f\left(k\right)\right)u_{d}\left(k\right),\\ y_{d}\left(k\right) & =C_{d}\left(f\left(k\right)\right)x_{d}\left(k\right)+v_{d}\left(k\right), \end{array}\right.$$
  where the vectors $x_{d}\left(k\right)\in R^{n_{d}}$, $u_{d}\left(k\right)\in R$, and $y_{d}\left(k\right)\in R$ denote the state, input, and output signal of the delay system, respectively. The matrices $G_{d}\left(f\left(k\right)\right)$, $H_{d}\left(f\left(k\right)\right)$, and $C_{d}\left(f\left(k\right)\right)$ denote the state matrix, the input matrix, and the output matrix, respectively.
  We denote the conceptual time-varying transfer operator of each delay block as $G_{d}\left(q^{-1},f\left(k\right)\right)$ with *q* the forward shift operator.
Step 4: As shown in Figure 5, based on the models of three delay blocks, the other cylinder pressure signals can be obtained by knowing the cylinder No. 1 pressure signal; thus, we can obtain a single-input single-output model between the cylinder No. 1 pressure signal and the vibration signal, and the model can be formulated as follows:
  (31) $$\left\{\begin{array}{cc} x_{s}\left(k+1\right) & =A_{s}\left(f\left(k\right)\right)x_{s}\left(k\right)+B_{s}\left(f\left(k\right)\right)P_{1}\left(k\right),\\ y(k) & =C_{s}\left(f\left(k\right)\right)x_{s}\left(k\right)+D_{s}P_{1}\left(k\right)+v_{s}\left(k\right), \end{array}\right.$$
  where $x_{s}\left(k\right)\in R^{n_{s}}$ denotes the state vector, the term $v_{s}\left(k\right)$ denotes the output error, and the matrices $A_{s}\left(f\left(k\right)\right)$, $B_{s}\left(f\left(k\right)\right)$, $C_{s}\left(f\left(k\right)\right)$, $D_{s}$, and the state vector $x_{s}\left(k\right)$ are given as follows:
  (32) $$A_{s}\left(f\left(k\right)\right)=\left(\begin{array}{cc} A & B\left(\begin{array}{ccc} 0 & 0 & 0\\ C_{d}\left(f\left(k\right)\right) & 0 & 0\\ 0 & C_{d}\left(f\left(k\right)\right) & 0\\ 0 & 0 & C_{d}\left(f\left(k\right)\right) \end{array}\right)\\ 0 & \left(\begin{array}{ccc} G_{d}\left(f\left(k\right)\right) & 0 & 0\\ H_{d}\left(f\left(k\right)\right)C_{d}\left(f\left(k\right)\right) & G_{d}\left(f\left(k\right)\right) & 0\\ 0 & H_{d}\left(f\left(k\right)\right)C_{d}\left(f\left(k\right)\right) & G_{d}\left(f\left(k\right)\right) \end{array}\right) \end{array}\right),$$
  (33) $$B_{s}\left(f\left(k\right)\right)={\left(\begin{array}{ccc} \left(\begin{array}{cccc} 1 & 0 & 0 & 0 \end{array}\right)B^{T} & {\left(H_{d}\left(f\left(k\right)\right)\right)}^{T} & 0 \end{array}\right)}^{T},$$
  (34) $$C_{s}\left(f\left(k\right)\right)=\left(\begin{array}{cc} C & D\left(\begin{array}{ccc} 0 & 0 & 0\\ C_{d}\left(f\left(k\right)\right) & 0 & 0\\ 0 & C_{d}\left(f\left(k\right)\right) & 0\\ 0 & 0 & C_{d}\left(f\left(k\right)\right) \end{array}\right) \end{array}\right),$$
  (35) $$D_{s}=D\left(\begin{array}{c} 1\\ 0\\ 0\\ 0 \end{array}\right),$$
  and
  (36) $$x_{s}\left(k\right)=\left(\begin{array}{c} x(k)\\ x_{d}\left(k\right)\\ x_{d}\left(k\right)\\ x_{d}\left(k\right) \end{array}\right),$$
  respectively.
Step 5: By augmenting the state of the model (29) with the state of the model (31), the augmented model $G_{a}$ can be obtained as follows:
  (37) $$\left\{\begin{array}{cc} x_{a}\left(k+1\right) & =A_{a}\left(f\left(k\right)\right)x_{a}\left(k\right),\\ y(k) & =C_{a}\left(f\left(k\right)\right)x_{a}\left(k\right)+v_{a}\left(k\right), \end{array}\right.$$
  where $x_{a}\left(k\right)\in R^{n_{a}}$ represents the state vector. The state matrix $A_{a}\left(f\left(k\right)\right)$, the output matrix $C_{a}\left(f\left(k\right)\right)$, and the state vector can be denoted as follows:
  (38) $$A_{a}\left(f\left(k\right)\right)=\left(\begin{array}{cc} A_{s}\left(f\left(k\right)\right) & B_{s}\left(f\left(k\right)\right)C_{p}\\ 0 & A_{p}\left(f\left(k\right)\right) \end{array}\right),$$
  (39) $$C_{a}\left(f\left(k\right)\right)=\left(\begin{array}{cc} C_{s}\left(f\left(k\right)\right) & D_{s}C_{p} \end{array}\right),$$
  and
  (40) $$x_{a}\left(k\right)=\left(\begin{array}{c} x_{s}\left(k\right)\\ x_{p}\left(k\right) \end{array}\right),$$
  respectively.
  $\left\{v_{a}\left(k\right)\right\}$ is assumed to be a scalar white noise process, of which the covariance function is $\sigma_{a}\delta_{kj}$, and the value of $\sigma_{a}$ is tunable.
Step 6: Based on the augmented model (37), the specific algorithm for reconstructing the cylinder No. 1 pressure signal is briefly displayed as a group of the following recursive equations:
  (41) $$\begin{array}{cc} {\hat{x}}_{a}^{-}\left(k\right) & =A_{a}\left(f\left(k\right)\right){\hat{x}}_{a}\left(k-1\right), \end{array}$$
  (42) $$\begin{array}{cc} {\hat{y}}^{-}\left(k\right) & =C_{a}\left(f\left(k\right)\right){\hat{x}}_{a}^{-}\left(k\right), \end{array}$$
  (43) $$\begin{array}{cc} e(k) & =y\left(k\right)-{\hat{y}}^{-}\left(k\right), \end{array}$$
  (44) $$\begin{array}{cc} {\hat{x}}_{a}\left(k\right) & ={\hat{x}}_{a}^{-}\left(k\right)+K\left(k\right)e\left(k\right), \end{array}$$
  (45) $$\begin{array}{cc} {\hat{x}}_{p}\left(k\right) & \leftarrow {\hat{x}}_{a}\left(k\right), \end{array}$$
  (46) $$\begin{array}{cc} {\hat{P}}_{1}\left(k\right) & =C_{p}{\hat{x}}_{p}\left(k\right), \end{array}$$
  where K(k) denotes the Kalman filter gain.
  It should be noted that under non-stationary operating conditions, a forgetting factor should be involved in the above Kalman filter. In addition to the cylinder No. 1 pressure estimate ${\hat{P}}_{1}\left(k\right)$, three other cylinder pressure signals can be simultaneously reconstructed using the model (30) of the delay block.

### 6.2. Sensor Fusion-Based Cylinder Pressure Reconstruction

**Figure 6:**
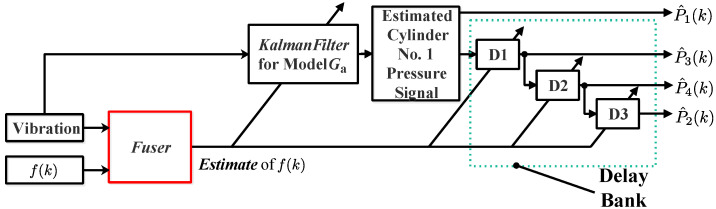
Cylinder pressure reconstruction framework using sensor-fused speed.

As shown in Figure 6, based on using the centralized sensor fusion technique, the cylinder pressure reconstruction approach is proposed. In Figure 6, it can be seen that the sensor-fused frequency $\hat{f}\left(k\right)$ is used instead of using the calculated instantaneous engine cycle frequency f(k). The corresponding algorithm is summarized in Algorithm 3.

**Algorithm 3:** Sensor-fused engine speed-based cylinder pressure reconstruction.

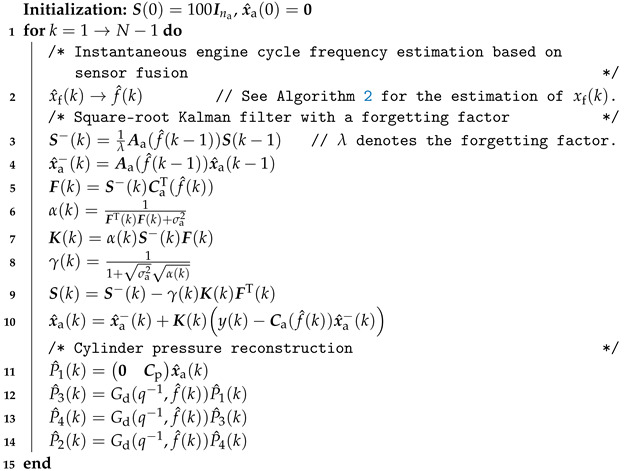



In Algorithm 3, the square-root Kalman filter including a forgetting factor is used to enhance numerical stability and accuracy and alleviate the effects from modeling error [35]. At each step *k*, the a priori error variance $P^{-}\left(k\right)=S^{-}\left(k\right){\left(S^{-}\left(k\right)\right)}^{T}$. Also, the a posteriori error variance $P\left(k\right)=S\left(k\right)S^{T}\left(k\right)$. F(k), α(k), and γ(k) are the intermediate variables.

In addition to the tunable parameters for the initialization of the Kalman filter, the remaining tunable parameters for Kalman filtering in Algorithm 3 are λ, Q, and R.

## 7. Experimental Studies

In this section, under the engine operating condition Z, we first use the sensor fusion-based engine speed estimation approach proposed in Section 5 to estimate the instantaneous engine cycle frequency f(k) under different values of Q and R, and then we compare the cylinder pressure reconstruction results using the proposed approach in Section 6.2 with the cylinder pressure reconstruction results from the approach presented in Section 6.1.

### 7.1. Sensor Fusion Results

In this paper, experimental studies use the operating condition around 2100 rpm to the operating condition around 3000 rpm; therefore, the lowest cutoff frequency and the highest cutoff frequency of the Butterworth bandpass filter can be set to 17.5 Hz and 25 Hz, respectively.

The performance of the extended Kalman filter derived in Section 5 can be affected by the covariance matrices Q and R [35]. In this section, the value of the covariance matrix Q is kept fixed, and the value of the covariance matrix R is tuned. Below, seven groups with a pair of Q and R are provided:

(47) $$\begin{array}{cc} \; & \mathrm{Group}\;A:\;Q=I_{5},\;R=\left(\begin{array}{cc} 1000 & 0\\ 0 & 1 \end{array}\right), \end{array}$$

(48) $$\begin{array}{cc} \; & \mathrm{Group}\;B:\;Q=I_{5},\;R=\left(\begin{array}{cc} 100 & 0\\ 0 & 1 \end{array}\right), \end{array}$$

(49) $$\begin{array}{cc} \; & \mathrm{Group}\;C:\;Q=I_{5},\;R=\left(\begin{array}{cc} 10 & 0\\ 0 & 1 \end{array}\right), \end{array}$$

(50) $$\begin{array}{cc} \; & \mathrm{Group}\;D:\;Q=I_{5},\;R=\left(\begin{array}{cc} 1 & 0\\ 0 & 1 \end{array}\right), \end{array}$$

(51) $$\begin{array}{cc} \; & \mathrm{Group}\;E:\;Q=I_{5},\;R=\left(\begin{array}{cc} 1 & 0\\ 0 & 10 \end{array}\right), \end{array}$$

(52) $$\begin{array}{cc} \; & \mathrm{Group}\;F:\;Q=I_{5},\;R=\left(\begin{array}{cc} 1 & 0\\ 0 & 100 \end{array}\right), \end{array}$$

(53) $$\begin{array}{cc} \; & \mathrm{Group}\;G:\;Q=I_{5},\;R=\left(\begin{array}{cc} 1 & 0\\ 0 & 1000 \end{array}\right). \end{array}$$

**Figure 7:**
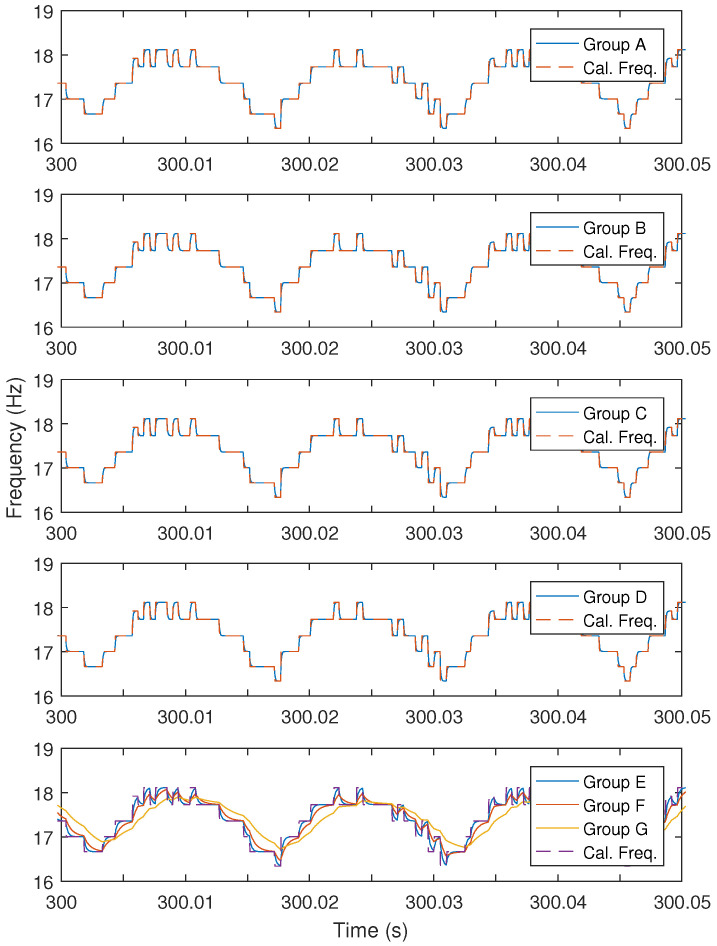
Sensor-fused frequencies and calculated frequency.

Based on seven groups from (47)–(53), seven sensor-fused instantaneous engine cycle frequencies can be obtained using the fuser shown in Figure 4. The fused frequencies are illustrated in Figure 7, and in the figure the calculated instantaneous engine cycle frequency (i.e., Cal. Freq.) is also shown.

In Figure 7, from Group A to Group D, the weightings on the vibration signal are larger than or equal to 1, it can be found that the fused instantaneous engine cycle frequencies are almost the same as the calculated engine cycle frequency. Contrarily, from Group E to Group G, as the weightings on the calculated engine cycle frequency becomes larger, the fused engine cycle frequencies become smoother.

### 7.2. Cylinder Pressure Reconstruction Results

In this section, the proposed cylinder pressure reconstruction approach in Section 6.2 is implemented to reconstruct the cylinder pressure under the non-stationary operating condition Z. In the experimental studies, the values of Q and R are tuned, while the forgetting factor λ and the covariance $\sigma_{a}$ are kept fixed, see Table 1.

Based on the approach in Section 6.2, the cylinder pressure reconstruction results under different fused instantaneous engine cycle frequencies are obtained, and in addition, the results are either compared with the reconstructed results obtained using the approach proposed by [26], in which the calculated instantaneous engine cycle frequency is directly used (see Figure 5). For approach validation, we use the reconstructed results of the cylinder No. 1 pressure signal because the pressure signals of the other three cylinders can be obtained using delay blocks.

Based on the cylinder pressure reconstruction results, the two combustion metrics pressure peak $P_{\max}$ and peak location $P_{\mathrm{loc}}$ are used for the evaluation of the cylinder pressure reconstruction approaches:

(1) $P_{\max}$ error:
  (54) $$e_{P_{\max}}=\frac{P_{\max}-{\hat{P}}_{\max}}{P_{\max}}\times 100\%.$$
(2) $P_{\mathrm{loc}}$ error:
  (55) $$e_{P_{\mathrm{loc}}}=P_{\mathrm{loc}}-{\hat{P}}_{\mathrm{loc}}.$$

The reason why the combustion metrics pressure peak $P_{\max}$ and peak location $P_{\mathrm{loc}}$ are used for evaluation of the cylinder pressure reconstruction results is that pressure peak $P_{\max}$ and peak location $P_{\mathrm{loc}}$ are two important parameters for the applications of engine in-cylinder pressure signals such as cylinder pressure-based internal combustion engine control [3,6].

**Figure 8:**
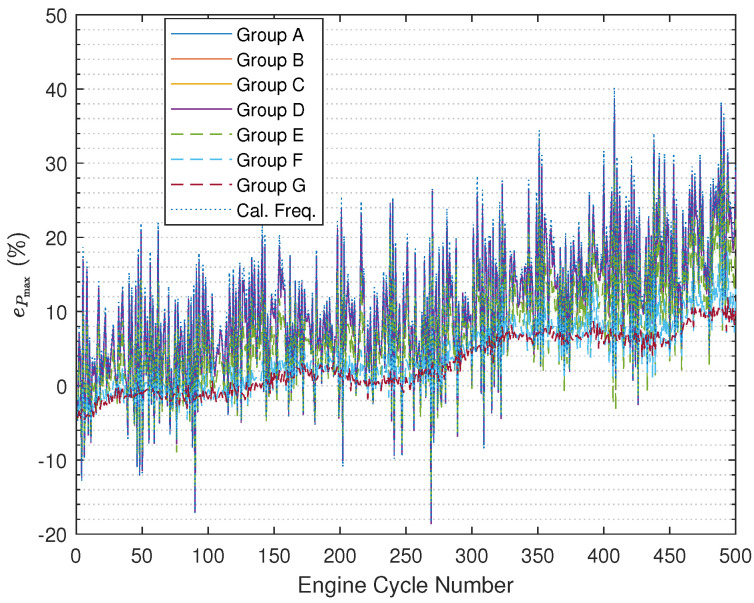
$P_{\max}$ error comparison.

**Figure 9:**
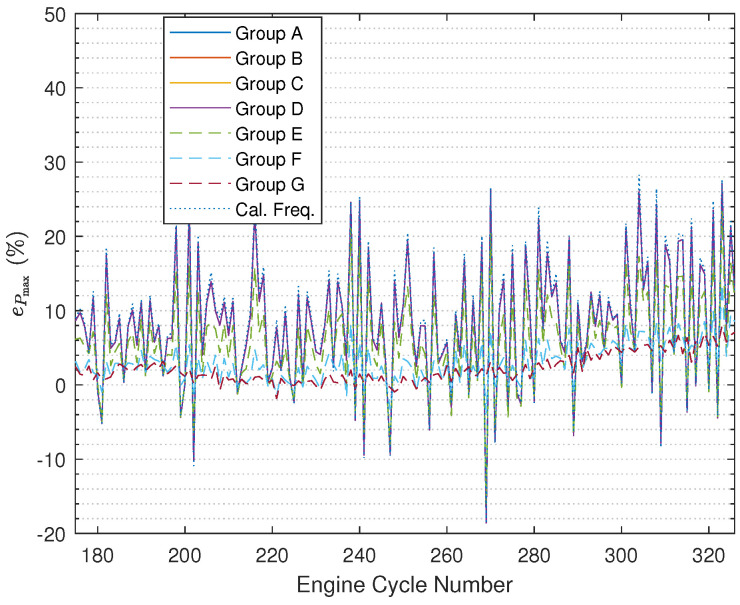
$P_{\max}$ error comparison (after zooming in).

**Figure 10:**
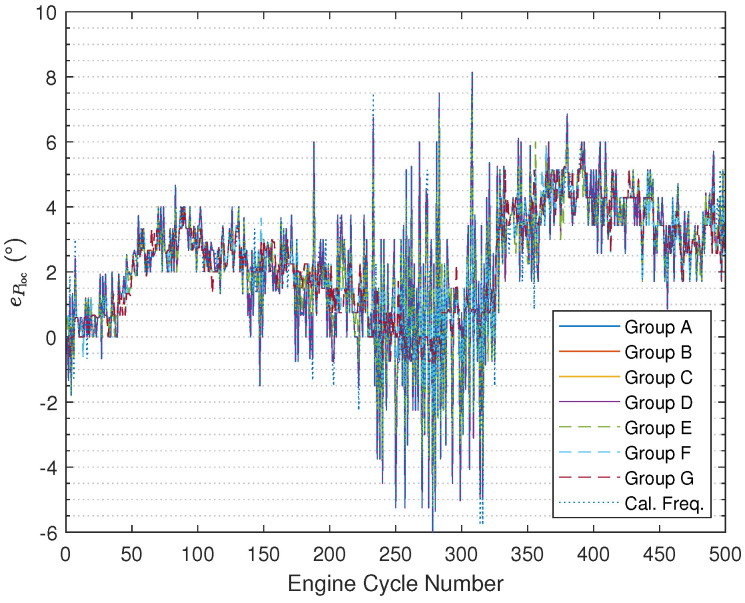
$P_{\mathrm{loc}}$ error comparison.

**Figure 11:**
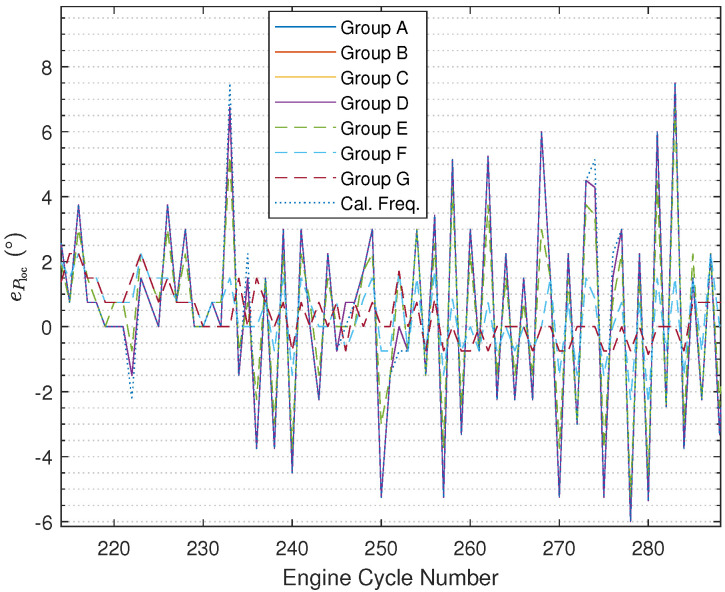
$P_{\mathrm{loc}}$ error comparison (after zooming in).

The results illustrated in Figure 8 (Figure 9 with zooming in) and Figure 10 (Figure 11 with zooming in) are obtained using (54) and (55), respectively. In the figures, the results are nearly the same under Group A, Group B, and Group C, and the curves overlap completely in the figures. In addition, it can be found that based on using the fused instantaneous engine cycle frequencies from Group E, Group F, and Group G, the errors (i.e., peak error and peak location error) are smaller than the errors obtained using the other instantaneous engine cycle frequencies (including the calculated engine cycle frequency), while the errors will get smaller if the ratio $\frac{R_{2}}{R_{1}}$ turns to larger, which indicates that when the weighting value $R_{2}$ for the calculated engine cycle frequency is larger, the filtered vibration signal $y_{b}\left(k\right)$ contributes more on the estimation of the engine cycle frequency. Furthermore, it can be also found that the errors under Group E, Group F, and Group G have slighter fluctuations than the errors under other groups. Moreover, it is interesting that if the ratio $\frac{R_{2}}{R_{1}}$ is smaller than 1 the errors will almost not change, the reason why this phenomenon occurs is that the small $\frac{R_{2}}{R_{1}}$ means we should believe the value of the calculated engine cycle frequency more such that the value of the fused engine cycle frequency is close to the value of the calculated engine cycle frequency. In addition, in the specified range of the engine crank angle, the peak error increases progressively while the trend of the peak location error does not change much.

**Figure 12:**
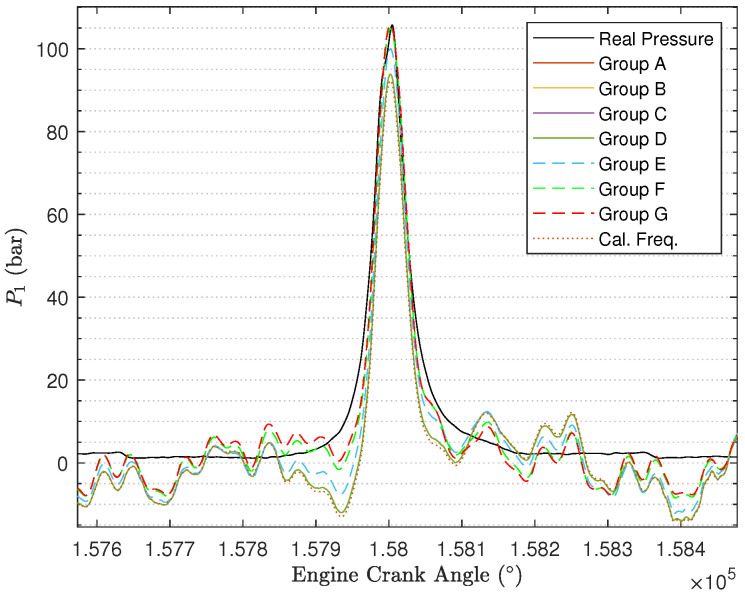
Reconstructed cylinder pressure signals under different values of Q and R.

**Figure 13:**
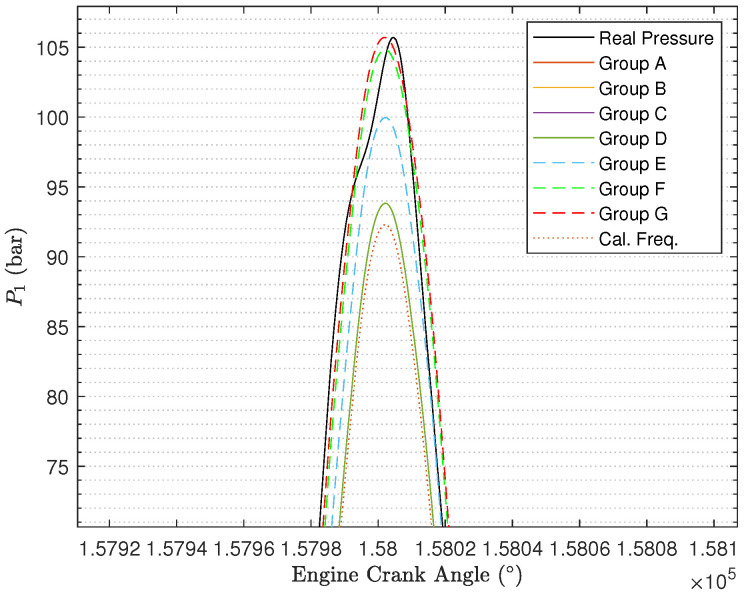
Reconstructed cylinder pressure signals under different values of Q and R (after zooming in).

It is still necessary to show the reconstructed cylinder pressure curves under different values of Q and R. In Figure 12 (Figure 13 with zooming in), we randomly select one engine cycle among 500 engine cycles to show the reconstructed cylinder pressure curves, and in the figure, the real cylinder pressure curve and the cylinder pressure signal reconstructed using the calculated engine cycle frequency are included for the purpose of better comparison. By comparing the cylinder pressure curves, it can be determined that the reconstructed cylinder pressure curves under Group E, Group F, and Group G are far better than the reconstructed cylinder pressure curves under Group A, Group B, Group C, and Group D. As the ratio $\frac{R_{2}}{R_{1}}$ becomes larger, the reconstructed cylinder pressure becomes better. Furthermore, the reconstructed cylinder pressure curves under Group A, Group B, Group C, and Group D are just slightly improved by comparison with the reconstructed cylinder pressure curve using calculated engine cycle frequency.

Above all, according to the cylinder pressure reconstruction results, it can display that sensor-fused engine speed has an effect on the cylinder pressure reconstruction accuracy, and when keeping the value of the matrix Q fixed, the larger the value of the ratio $\frac{R_{2}}{R_{1}}$, the higher the accuracy of the cylinder pressure reconstruction.

## 8. Conclusions and Perspectives

In this paper, a sensor fusion-based engine speed estimation approach is proposed, based on which a sensor-fused engine speed-based cylinder pressure reconstruction approach is proposed. The results of the experimental studies show the effectiveness of the proposed cylinder pressure reconstruction approach. A sensor-fused engine speed with larger $\frac{R_{2}}{R_{1}}$ can improve the accuracy of the reconstructed cylinder pressure. The sensor-fused engine speed shows its prospects in the enhancement of cylinder pressure reconstruction performance. In the future, we will build a more accurate model between cylinder pressure and vibration, and involve it in the proposed approach of this paper to improve the cylinder pressure reconstruction accuracy. Additionally, even though we know that λ should be smaller than 1, the best value of the tunable variable λ for the cylinder pressure reconstruction will be explored.

## Figures and Tables

**Table 1:** Values of the variables.

Parameter	Value
λ	0.9995
$\sigma_{a}$	0.00001

## Data Availability

The data presented in this study are available on request from the corresponding author.
